# Identification of a Novel Signaling Pathway and Its Relevance for GluA1 Recycling

**DOI:** 10.1371/journal.pone.0033889

**Published:** 2012-03-21

**Authors:** Guiscard Seebohm, Sebastian Neumann, Carsten Theiss, Tanja Novkovic, Elaine V. Hill, Jeremy M. Tavaré, Florian Lang, Michael Hollmann, Denise Manahan-Vaughan, Nathalie Strutz-Seebohm

**Affiliations:** 1 Department of Biochemistry I - Cation Channel Group, Ruhr University Bochum, Bochum, Germany; 2 Department of Biochemistry II – Molecular Neurobiochemistry, Ruhr University Bochum, Bochum, Germany; 3 Department of Anatomy and Molecular Embryology, Ruhr University Bochum, Bochum, Germany; 4 Department of Neurophysiology, Ruhr University Bochum, Bochum, Germany; 5 School of Biochemistry, University of Bristol, Bristol, England; 6 Department of Physiology, University Tuebingen, Tuebingen, Germany; 7 Department of Biochemistry I - Receptor Biochemistry, Ruhr University Bochum, Bochum, Germany; Institut National de la Santé et de la Recherche Médicale, France

## Abstract

We previously showed that the serum- and glucocorticoid-inducible kinase 3 (SGK3) increases the AMPA-type glutamate receptor GluA1 protein in the plasma membrane. The activation of AMPA receptors by NMDA-type glutamate receptors eventually leads to postsynaptic neuronal plasticity. Here, we show that SGK3 mRNA is upregulated in the hippocampus of new-born wild type Wistar rats after NMDA receptor activation. We further demonstrate in the *Xenopus* oocyte expression system that delivery of GluA1 protein to the plasma membrane depends on the small GTPase RAB11. This RAB-dependent GluA1 trafficking requires phosphorylation and activation of phosphoinositol-3-phosphate-5-kinase (PIKfyve) and the generation of PI(3,5)P_2_. In line with this mechanism we could show PIKfyve mRNA expression in the hippocampus of wild type C57/BL6 mice and phosphorylation of PIKfyve by SGK3. Incubation of hippocampal slices with the PIKfyve inhibitor YM201636 revealed reduced CA1 basal synaptic activity. Furthermore, treatment of primary hippocampal neurons with YM201636 altered the GluA1 expression pattern towards reduced synaptic expression of GluA1. Our findings demonstrate for the first time an involvement of PIKfyve and PI(3,5)P_2_ in NMDA receptor-triggered synaptic GluA1 trafficking. This new regulatory pathway of GluA1 may contribute to synaptic plasticity and memory.

## Introduction

Excitatory neurotransmission has been thoroughly described at hippocampal synapses, especially those between Schaffer collaterals and dendrites of CA1 pyramidal neurons [1]. At these synapses, different subtypes of glutamate receptors, chiefly AMPA and NMDA receptors, coexist. AMPA-type glutamate receptors are rapidly shuttled into and out of synapses to strengthen or weaken their function [1], [2], [3], [4], [5]. At resting membrane potentials, synaptic glutamate evokes an excitatory postsynaptic current (EPSC) that is mediated almost entirely by AMPA receptors. Depolarization relieves the Mg^2+^ blockade of the NMDA receptors, hence subsequent EPSCs contain contributions from both AMPA and NMDA receptors.

At hippocampal synapses, a large increase in intracellular calcium concentration mediated by NMDA receptors activates kinases, enhances activity of synaptic AMPA receptors, and triggers long term potentiation (LTP) [1], [5], [6], [7].

In the CA1 subfield of the hippocampus, a silent synapse is defined as a synapse in which EPSCs are absent at the resting membrane potential but become apparent on depolarization. Silent synapses are thought to reflect the functional presence of NMDA but not AMPA receptors. Because only AMPA receptors can conduct current at the resting membrane potential, the absence of functional postsynaptic AMPA receptors renders a synapse “silent”. Interestingly, manipulations designed to trigger LTP in the hippocampus also “unsilence” these silent CA1 synapses [3]. Candidate signaling molecules involved in this complex regulatory mechanism of synaptic plasticity include SGK, which has been shown before to regulate AMPA receptor plasma membrane expression [8]. Other candidate proteins that may affect GluA1 receptor trafficking include RAB family proteins, which are GTPases involved in vesicle cycling [9]. RAB5, a monomeric GTPase of the Ras superfamily, has been implicated in the regulation of early steps in the endocytic pathway, whereas the RAB11 GTPase is localized at the trans-Golgi network, post-Golgi vesicles, and the recycling endosome [10], [11]. Mammalian cells and *Xenopus laevis* oocytes possess and use highly conserved RAB-dependent trafficking pathways [12]. Endocytosis by RAB5 and plasma membrane-directed transport by RAB11 participate in the regulation of CFTR chloride channels [13] and the glucose transporter GLUT4 [14], [15]. The RAB-dependent regulation of GLUT4 also involves the phosphoinositol-3-phosphate-5-kinase (PIKfyve) that generates the phosphatidylinositol PI(3,5)P_2_ [16]. PIKfyve is stimulated by protein kinase B, a close relative of SGK3, phosphorylating serine and threonine residues within a similar core consensus sequence (RXRXX[S/T]) [17]. We here identify a novel mechanism involving NMDA receptor-triggered, SGK3-dependent stimulation of PIKfyve with subsequent formation of PI(3,5)P_2_, which modulates RAB11A-facilitated vesicle transport to the plasma membrane, leading to an increased abundance of GluA1 receptor subunits in the plasma membrane. We suggest that this novel mechanism plays a role in the dynamic regulation of GluA1 at synapses.

## Results

### SGK3 mRNA is upregulated in hippocampus after NMDA receptor activation

**Figure 1:**
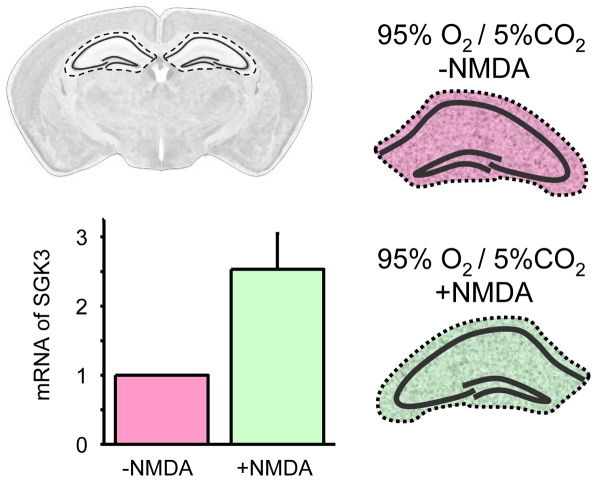
SGK3 mRNA expression in hippocampus after pharmacological NMDA receptor stimulation. mRNA levels of SGK3 before and after incubation of hippocampal slices with 10 µM NMDA for 30 minutes, determined by quantitative RT-PCR. The expression data from hippocampus were normalized to the expression of the housekeeping gene β-actin. The quantitative RT-PCR revealed a 2.5±0,52 fold increase in SGK3 mRNA in NMDA-treated compared to non-treated hippocampal slices. Number of experiments n = 4.

We have previously shown that SGK3 increases glutamate-induced GluA1 receptor currents. As a first step to evaluate whether SGK3 plays a regulatory role in dynamic processes at the glutamatergic synapses, we determined the mRNA level of SGK3 in hippocampus after pharmacological NMDA receptor stimulation. To examine the SGK3 mRNA level, hippocampal slices were incubated with 10 µM NMDA for 30 minutes, total RNA was isolated and quantitative RT-PCR performed. This analysis revealed a 2.5fold increase of SGK3 mRNA in NMDA-treated compared to non-treated hippocampal slices (Figure 1).

### PIKfyve as a potential candidate molecule for SGK3-dependent GluA1 regulation

**Figure 2:**
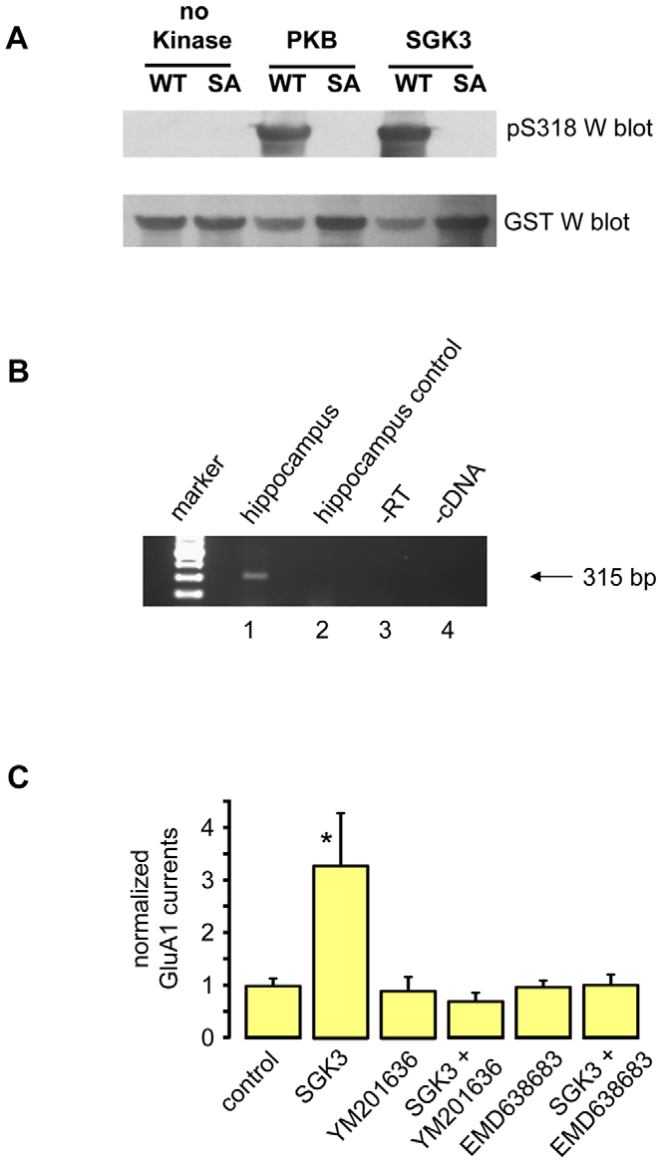
PIKfyve expression, phosphorylation, and function. (**A**) PIKfyve is phosphorylated at Ser318 by SGK3 and at Ser318 by PKB. Purified recombinant GST-tagged wild-type (WT) or S318A mutant (SA) of PIKfyve was subjected to Western blotting. Blots were incubated with rabbit anti-GST antibody (PIKfyve; lower panel), followed by stripping and reprobing with a rabbit anti-PIKfyve antibody specific for phosphoserine 318 (apS318; top panel). (**B**) RT-PCR demonstrating that PIKfyve is expressed in hippocampus. Lane 1: cDNA from hippocampus, the two primers bind in exons 19 and 20, respectively, and amplify a 315 bp fragment of PIKfyve; lane 2: control reaction to exclude genomic contamination; lane 3: control reaction without reverse transcriptase; lane 4: control reaction without cDNA. (**C**) The PIKfyve inhibitor YM201636 and SGK inhibitor EMD638683 suppress the upregulating effect of SGK3 on GluA1 currents. GluA1 current amplitudes in oocytes expressing GluA1. Acute injection of purified active SGK3 protein led to an increase in GluA1 currents. The effects of YM201636 and EMD638683 on GluA1 currents were measured in oocytes before and after acute injection of SGK3 protein. Significant change to GluA1 control (p = 0.013) is indicated by *. Numbers of oocytes were n = 7–36.

**Figure 3:**
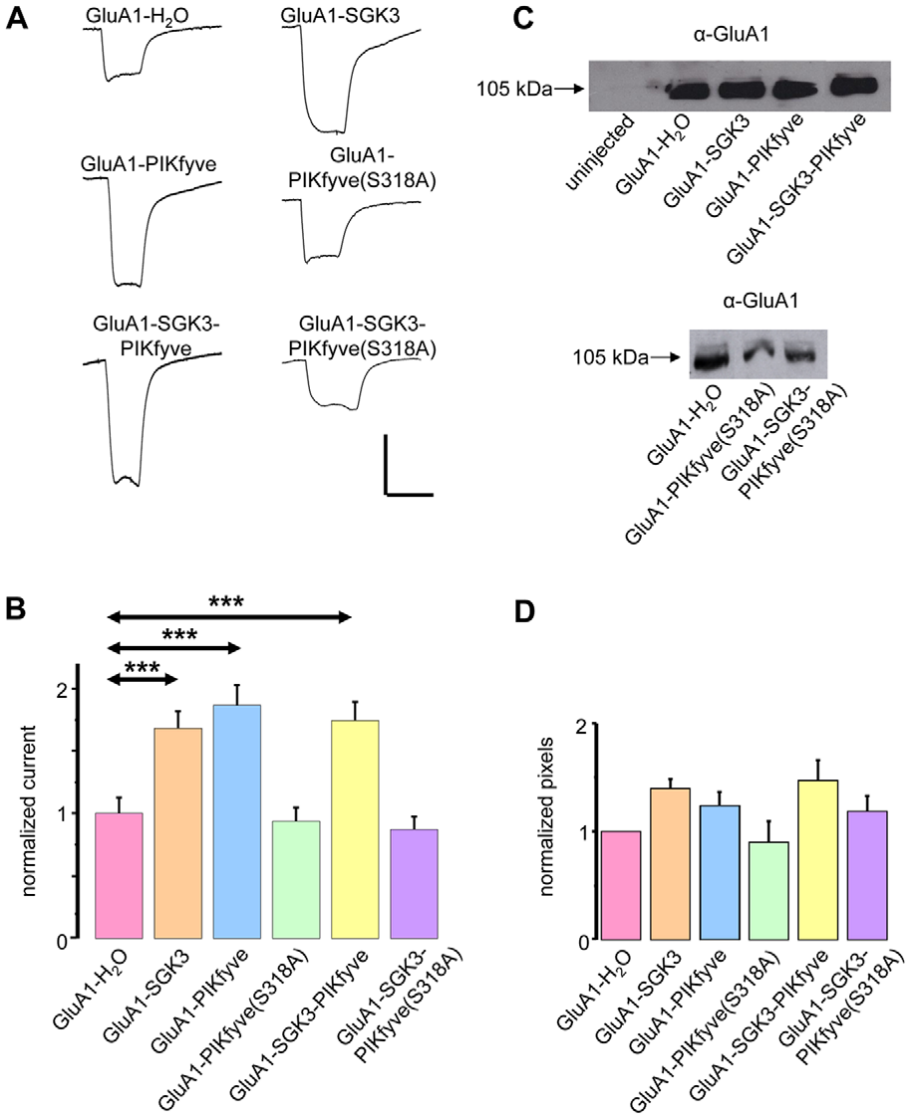
GluA1 function is increased by SGK3 and PIKfyve. (**A**) Representative current traces measured in *Xenopus* oocytes in response to superfusion with 300 µM glutamate plus 100 µM cyclo-thiacide. All currents were measured at −70 mV. Vertical scale-bar, 0,5 µA; horizontal bar, 4 s. (**B**) GluA1 current amplitudes in oocytes expressing GluA1, or combinations of GluA1 with SGK3, the inactive form of PIKfyve (PIKfyve(S318A)), or wild type PIKfyve. Numbers of oocytes are n = 20–30. Significant changes (p<0.001) are indicated by *** (p = 0.00049; 0.00036; 0.000073, respectively). (**C**) Representative samples including controls from uninjected oocytes were biotinylated to isolate plasma membrane GluA1, then separated on an SDS gel, Western-blotted and probed with a primary rabbit anti-GluA1 antibody. The GluA1 protein has an apparent molecular weight of ∼105 kDa. (**D**) Bar graph showing relative abundance of GluA1 plasma membrane protein. The band intensity was quantified by densitometric analysis using the software Scion image.

In earlier studies, we showed that phosphoinositol-3-phosphate-5-kinase (PIKfyve) is involved in SGK1-dependent regulation of transporters and channels (e.g. EAAT 2, −3, −4, CFTR, ClC2) [18], [19], [20], [21], [22], and that PIKfyve is phosphorylated on Ser318 by SGK1 [23]. However, SGK3 was not tested as a possible PIKfyve-targeting kinase. Since among the three SGK isoforms SGK3 provides the most prominent stimulatory effect on GluA1 [8], it was of special interest to investigate whether SGK3 phosphorylates PIKfyve similar to SGK1. Here, we selectively analyzed the putative phosphorylation site S318 in PIKfyve for phosphorylation by SGK3 by using a specific antibody directed against pS318. The Western blot in Figure 2A demonstrates that SGK3 as well as PKB phosphorylate PIKfyve at position S318, thereby indicating that PIKfyve could be a physiological target of SGK3. However, expression of PIKfyve in brain, especially in hippocampus, where GluA1 and SGK3 are expressed, has not yet been demonstrated. Here, we show mRNA expression of PIKfyve in mouse hippocampal tissue by performing RT-PCR (Figure 2B). To further analyze the possible modulatory role of PIKfyve in the SGK3-dependent regulation of GluA1, we recorded current amplitudes from *Xenopus* oocytes expressing either GluA1 or GluA1 plus SGK3 before and after incubation with the PIKfyve inhibitor YM201636 or SGK inhibitor EMD638683 (Figure 2C). Both inhibitors fully abolished the stimulating effect of SGK3. The SGK3 effects on GluA1 channel currents were mimicked by overexpression of PIKfyve and abrogated by site-directed mutagenesis that replaced PIKfyve Ser318 by Ala, indicating that PIKfyve is indeed a downstream target of SGK3 (Figure 3). The membrane abundance of GluA1 was determined by performing an oocyte plasma membrane biotinylation assay with subsequent SDS gel electrophoresis and Western blotting. For quantification, we calculated the mean intensity from three different blots each of which had been normalized to GluA1 expressed alone. As illustrated in Figure 3C and D, the protein membrane abundance of GluA1 is increased in oocytes expressing GluA1 together with SGK3 and PIKfyve as compared to the GluA1 protein abundance in oocytes expressing GluA1 alone, suggesting modifications in GluA1 trafficking by SGK3/PIKfyve.

### Plasma membrane-directed trafficking of GluA1 proteins requires functional RAB11, phosphorylation-mediated activation of PIKfyve as well as the generation of PI(3,5)P_2_

**Figure 4:**
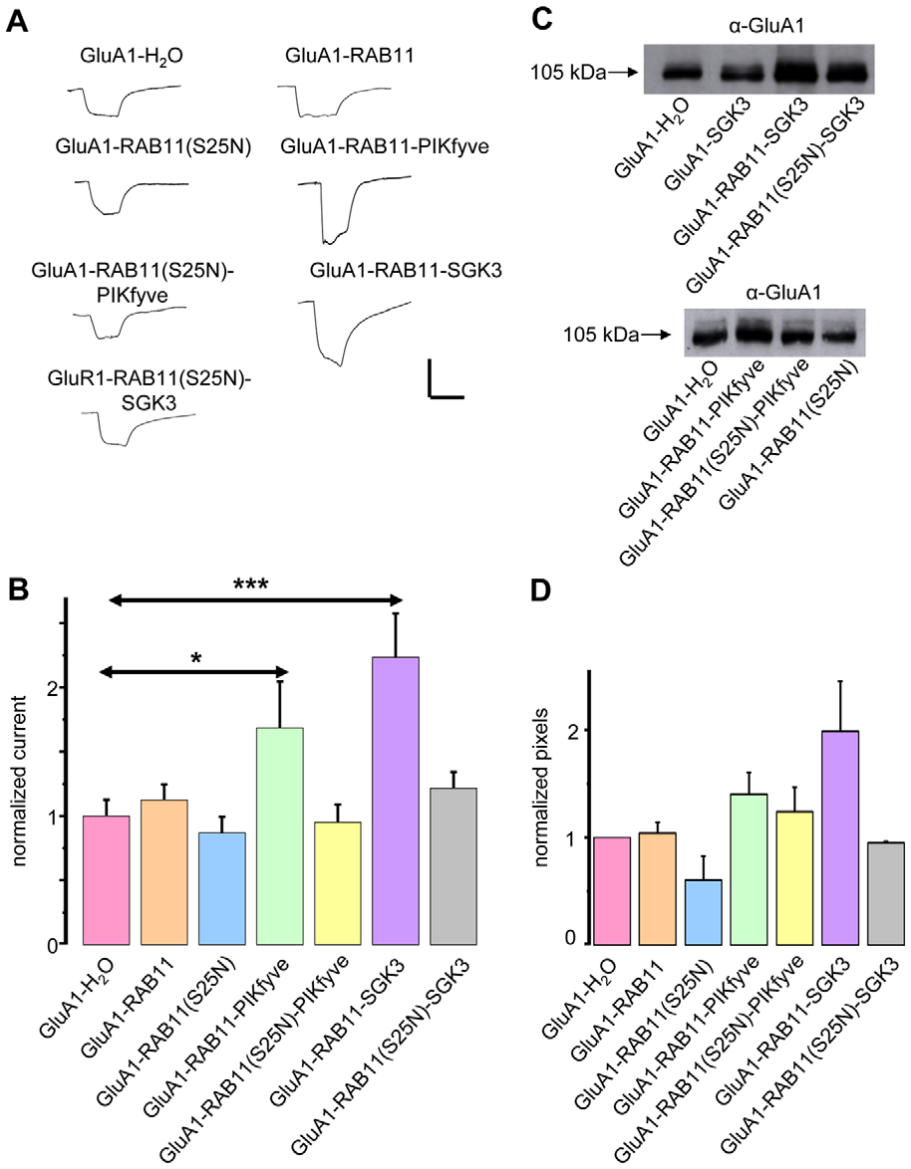
Regulation of GluA1 by SGK3 and PIKfyve is Rab11-dependent. (**A**) Representative current traces measured in *Xenopus* oocytes in response to superfusion with 300 µM glutamate plus 100 µM cyclo-thiacide. All currents were measured at −70 mV. Vertical scale-bar, 0,5 µA; horizontal bar, 4 s. (**B**) GluA1 current amplitudes in oocytes expressing GluA1, or combinations of GluA1 with Rab11, the dominant negative form of Rab11 (Rab11DN), PIKfyve, or SGK3. Numbers of oocytes are n = 15–30. Significant changes (p<0.001, p<0.05) are indicated by *** (p = 0.00015) and * (p = 0.036), respectively. (**C**) Representative samples including controls from uninjected oocytes were biotinylated to isolate plasma membrane GluA1, then separated on an SDS gel, Western-blotted and probed with a primary rabbit anti-GluA1 antibody. The GluA1 protein has an apparent molecular weight of ∼105 kDa. (**D**) Bar graph showing relative abundance of GluA1 plasma membrane protein. The band intensity was quantified by densitometric analysis using the software Scion image.

**Figure 5:**
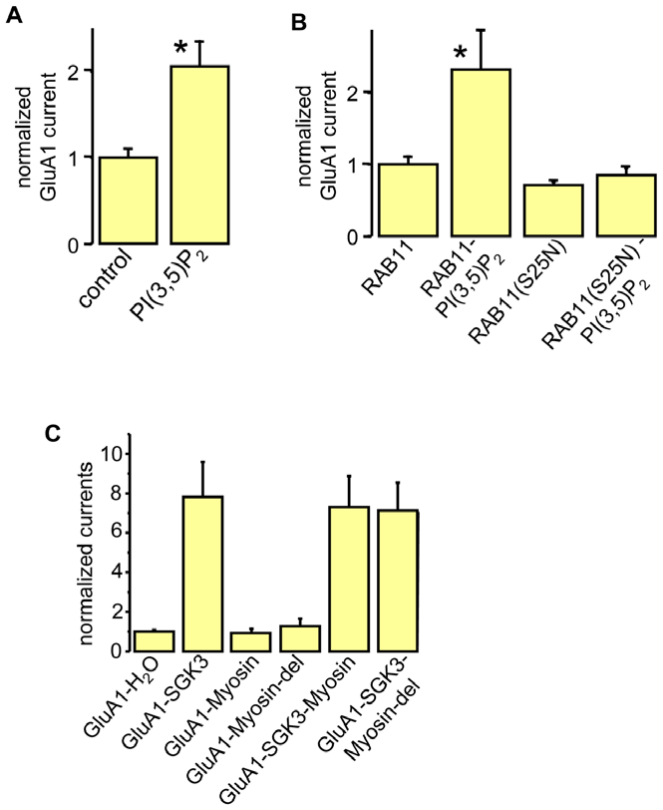
The SGK3-dependent trafficking of GluA1 proteins requires functional RAB11 and the generation of PI(3,5)P_2_, but is myosin-independent. (**A**) GluA1 current amplitudes in oocytes before and after acute injection of a water-soluble analog of PI(3,5)P_2_. Significant change (p<0.05) is indicated by * (p = 0.0015). (**B**) GluA1 current amplitudes in oocytes expressing GluA1 or combinations of GluA1 with Rab11 or the dominant negative form of Rab11 (Rab11DN), before and after acute injection of a water-soluble analog of PI (3,5)P_2_. Numbers of oocytes are n = 7–28. Significant change (p<0.05) is indicated by * (p = 0.025). (**C**) GluA1 current amplitudes in oocytes expressing GluA1 or combinations of GluA1 with SGK3, myosin Vb, or the mutated form of myosin Vb (myosin del). Number of oocytes are n = 14–28.

We have shown before that the increase in GluA1 current amplitudes by SGK3 is a result of enhanced membrane expression [8]. It is known that, among other proteins, the GTPase Rab11 regulates vesicle transport of AMPA receptors [4], [9], [24], [25]. Therefore, we tested the importance of Rab11 for the SGK3-dependent regulation of GluA1. We addressed this issue by coexpression studies in *Xenopus* oocytes. As shown in Figure 4, Rab11 itself had no effect on GluA1 current amplitudes (Figure 4A, B) nor on GluA1 plasma membrane expression (Figure 4C, D). However, when a dominant negative mutant of Rab11 (Rab11(S25N)) was coexpressed with GluA1, the stimulation by PIKfyve or SGK3 was abrogated, suggesting that Rab11 is a downstream effector of the described SGK3 cascade (Figure 4). In theory, PIKfyve-dependent regulation of Rab11 could involve direct phosphorylation of Rab11 by PIKfyve, or stimulation of Rab11 by the PIKfyve product, PI(3,5)P_2_. Thus, we performed an acute injection of a water-soluble analog of PI(3,5)P_2_ into oocytes overexpressing GluA1 either alone (Figure 5A), or together with Rab11 or Rab11(S25N) (Figure 5B). The acute injection of the lipid PI(3,5)P_2_ led to a significant increase in GluA1 current amplitudes for oocytes either expressing GluA1 or GluA1 plus Rab11. For oocytes expressing GluA1 plus Rab11(S25N) no stimulatory effect was observed, suggesting that the product of PIKfyve, PI(3,5)P_2_, modulates Rab11-dependent trafficking of GluA1. As mentioned above, others have shown a regulatory role of Rab11 on GluA1 [25]. In one study, Wang et al. found that myosin Vb binds to Rab11/Rab11FIP2 (Rab11-family interacting protein 2). Myosin Vb anchors the complex to the cytoskeleton. We thus tested whether our newly discovered regulatory cascade of GluA1 also involves myosin Vb, or if the SGK3 cascade operates in a parallel modulatory pathway. To this end we coexpressed GluA1 with myosin Vb, or a myosin Vb mutant (myosin del) with inhibited ability to bind to Rab11/Rab111FIP2, either with or without SGK3. The results shown in Figure 5C indicate that the SGK3 effect on GluA1 trafficking does not depend on myosin Vb, at least not in this heterologous expression system. We therefore conclude that the SGK3-stimulated Rab11-dependent GluA1 regulation described here is different from that discovered by Wang *et al.*

### Decrease of synaptic GluA1 receptors after treatment with a PIKfyve inhibitor

**Figure 6:**
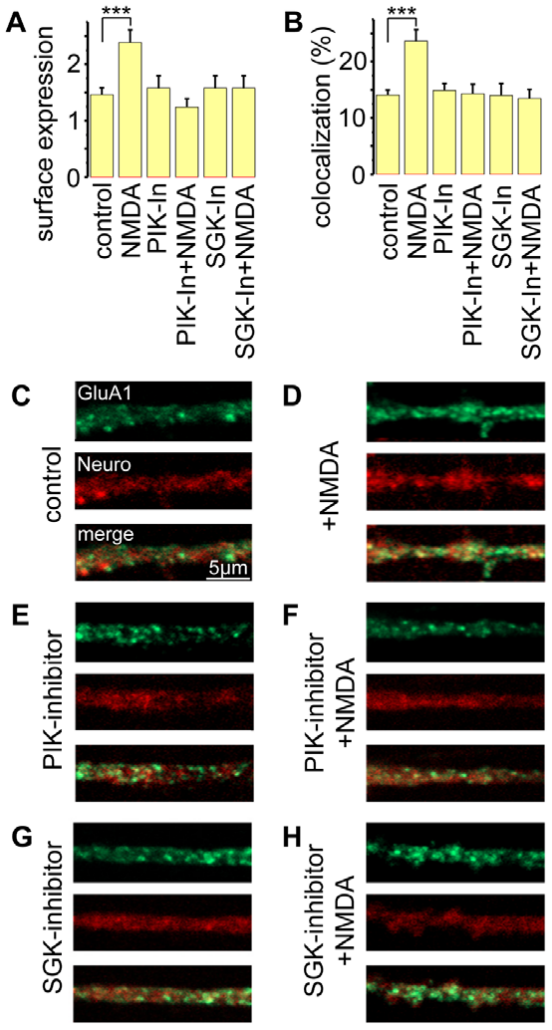
Reduced GluA1 expression after treatment with an SGK inhibitor or a PIKfyve inhibitor. (**A**, **B**) Statistical analysis of GluA1 surface expression and colocalization of GluA1 with neuroligin revealed increased surface and synaptic GluA1 expression after treatment with NMDA. This effect is abolished by SGK or PIKfyve inhibition. Control = untreated, NMDA = incubated with NMDA, PIK-In = treated with PIKfyve inhibitor, SGK-In = treated with SGK inhibitor. (C–H) Representative confocal images of dendrites stained with GluA1 (green) and neuroligin (red –Neuro) in controls versus NMDA-treated neurons under different conditions: (**C**) control versus (**D**) NMDA, (**E**) PIKfyve inhibitor (PIK inhibitor), (**F**) PIKfyve inhibitor and NMDA (PIK inhibitor+NMDA), (**G**) SGK inhibitor (SGK inhibitor), (**H**) SGK inhibitor and NMDA (SGK inhibitor+NMDA). The magnification is equal for all images - scale bar indicates 5 µm. Number of images analyzed n = 10–15, obtained from independent neuron culture preparations.

After showing modulation of GluA1 trafficking by SGK3, we explored whether the synaptic or extrasynaptic fraction of GluA1 subunits is affected via this signaling cascade, upon NMDA receptor activation. Therefore, we analyzed cultured hippocampal neurons for GluA1 expression under various conditions of pharmacological NMDA receptor activation. To this end neurons were stained with primary anti-GluA1 antibody and anti-neuroligin-1 antibody as postsynaptic marker for non-permeabilized neurons (Figure 6), or anti-PSD95 for permeabilized neurons (Figure S1). Utilizing confocal laser scanning microscopy non-permeabilized neurons were analyzed by counting neuroligin-stained as well as GluA1-labeled spines and calculating the percentage of overlapping expression of neuroligin with GluA1 with the aid of the Zeiss physiology kit (Figure 6C). Hippocampal neurons were incubated with 10 µM NMDA and 3 µM PIKfyve inhibitor (YM201636), or 30 µM SGK inhibitor (EMD638683), either alone or together for 20 minutes each, prior to fixation. As a control we used untreated neurons. Incubation with NMDA clearly increased overlapping expression of neuroligin or PSD95 with GluA1 at synapses (Figure 6, Figure S1). Coincubation with NMDA and PIKfyve inhibitor or SGK inhibitor reduced synaptic expression of GluA1. These observations suggest a regulatory role for an NMDA receptor-triggered SGK3-PIKfyve-dependent cascade in synaptic expression of GluA1 receptors.

### Basal synaptic transmission is reduced by a PIKfyve inhibitor

**Figure 7:**
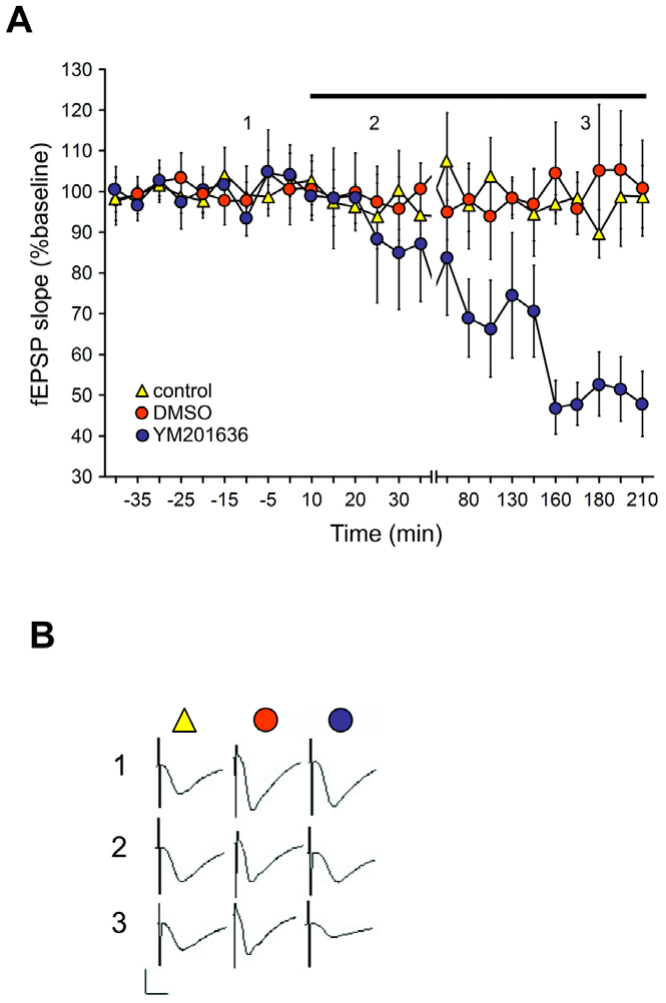
PIKfyve inhibition results in depression of hippocampal basal synaptic transmission. (**A**) Basal synaptic transmission was recorded in the CA1 region of hippocampal slices. Control (non-treated) hippocampi, or hippocampi treated with DMSO showed stable evoked responses throughout the 210 min recording period. Treatment with the PIKfyve inhibitor (YM201636) elicited a depression of evoked responses that became evident 40 min after drug application. Line-breaks indicate changes in time-scale. Black bar indicates DMSO or drug application. (**B**) Analog traces representing evoked potentials obtained in control slices or in slices in the presence of DMSO or YM201636, at the time-points indicated in the graph. Vertical scale-bar, 3 mV; horizontal bar, 3 ms.

To explore the consequences of the observed signaling cascade for evoked potentials, we performed electrophysiological experiments on hippocampal slices to analyze basal synaptic transmission under normal conditions and during incubation with a PIKfyve inhibitor. We evoked field excitatory postsynaptic potentials (fEPSP) by stimulating the Schaffer collaterals and recording from the CA1 Stratum radiatum. Control responses (n = 8) and responses obtained in the presence of DMSO (vehicle, n = 8) were stable throughout the recording period (Figure 7). Basal synaptic transmission was significantly reduced, however, by the PIKfyve inhibitor YM201636 (n = 6). (ANOVA: F(1,24) = 50,56, p<0.001). This observation further supports a regulatory role of PIKfyve on synaptic glutamate receptor expression.

## Discussion

**Figure 8:**
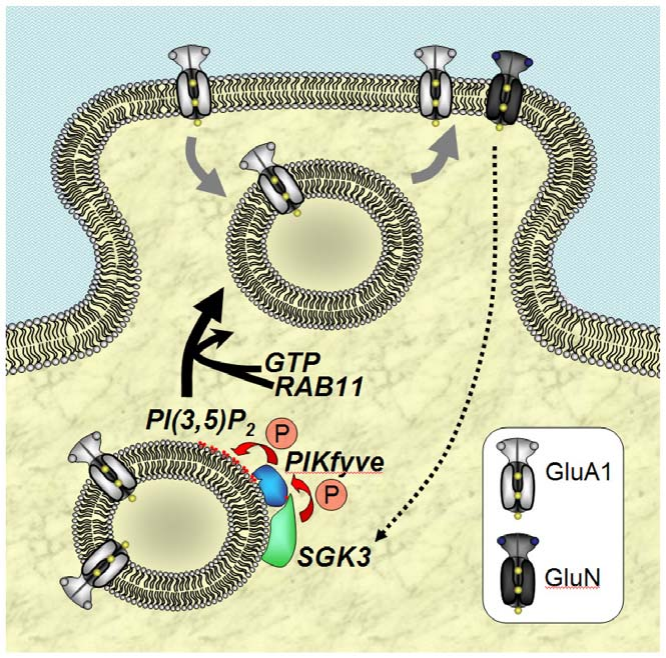
Postulated model of SGK3-dependent regulation of GluA1. Upon stimulation of NMDA receptors SGK3 is transcriptionally upregulated. SGK3 in turn phosphorylates/activates PIKfyve, which leads to local production of PI(3,5)P_2_ in PIKfyve-containing recycling vesicles. PI(3,5)P_2_ in turn stimulates Rab11-dependent plasma membrane-directed trafficking of GluA1-containing vesicles.

In the past few years, it has become increasingly clear that dynamic regulation of AMPA-type receptors at the synapse plays a critical role in alterations of synaptic strength (for review: [2], [3]). AMPA receptors undergo constant trafficking in and out of synapses by a combination of endocytotic retrieval, membrane-directed transport, and lateral diffusion in the membrane. Although the underlying mechanisms are far from being fully understood, it is safe to state that all three processes participate in receptor exchange at synapses at rest and during various forms of plasticity [26]. The trafficking of AMPA receptors can occur within minutes [27]. Protein phosphorylation plays a central role in controlling AMPA receptor expression at the synapse and in regulating synaptic strength [28], [29]. The multiple signaling pathways underlying the regulation of AMPA receptor trafficking include the phospatidylinositol-3-kinase (PI3-kinase) pathway [30]. Downstream targets of the PI3-kinase include the phosphoinositide-dependent kinases PDK1, protein kinase B as well as the serum- and glucocorticoid-inducible kinase isoforms including SGK3. SGK3 is abundantly expressed in the brain and upregulates GluA1 plasma membrane expression [8]. Our findings suggest a novel principle for synaptic regulation which involves modulation of GluA1 expression levels by SGK3 as a key feature. Recently, increased attention has been directed towards PIKfyve, whose importance for regulation of ion channel trafficking becomes more and more apparent [17], [18], [19], [20], [21], [22], [23]. PIKfyve is phosphorylated at position Ser318 by SGK1 [23] and, as shown in this study, is also phosphorylated at this site by SGK3. Phosphorylation of Ser318 of PIKfyve leads to its activation and increased PI(3,5)P_2_ production [17]. The effects of SGK3 on GluA1 current amplitudes were mimicked by overexpression of PIKfyve and abrogated by site-directed mutagenesis that replaced Ser318 by Ala. Furthermore, GluA1 receptor currents were enhanced in response to injection of PI(3,5)P_2_. The effect of SGK3 on GluA1 was not additive to that of PIKfyve, indicating that PIKfyve is indeed a downstream target of SGK3 (Figure 8). The observation that PI(3,5)P_2_ plays a regulatory role in this cascade is especially interesting, as the role of PI(3,5)P_2_ in regulation of glutamate receptors has never before been explored. However, it has been reported by Arendt *et al.* that synthesis and availability of phosphatidylinositol-(3,4,5)-trisphosphate (PI(3,4,5)P_3_) at the postsynaptic terminal is a precondition for sustained synaptic function by maintaining AMPA receptor clustering in hippocampal neurons [31]. PIP_3_ downregulation led to a depression of synaptic transmission and impaired PSD-95 accumulation in spines. It remains to be elucitated if the PI(3,4,5)P_3_ –dependent regulation of AMPA receptors, as observed by Arendt et al., underlies the same regulatory mechanism observed by us for GluA1, a mechanism which, however, is PI(3,5)P_2_–dependent. Our experiments with myosin Vb indicate myosin-independent regulation and thus a different regulatory mechanism than shown by Wang et al. (2008).

The mechanism proposed in this study is based on the observation that NMDA receptor activation in mouse hippocampi triggers transcriptional stimulation of SGK3. It seems that the relevant phospholipid PI(3,5)P_2_ is selectively and efficiently produced intracellulary at recycling vesicles by PIKfyve [32], [33]. The specific localization of PIKfyve at these recycling vesicles enables production of this rare form of PIP_2_ specifically at these vesicles. The fact that inhibition of SGK3 and PIKfyve were both able to inhibit the translocation to the plasma membrane suggests a key role for SGK3 and PIKfyve in this scenario (Figure 8). Although, theoretically, PI(3,5)P_2_ can also be produced by PI3K *in vitro*, PI3 kinase has not been reported being expressed in these recycling vesicles. Therefore, PI3K involvement in the mechanism described here could be negligible.

In summary, our observations suggest that NMDA receptor-triggered SGK3 mRNA upregulation, SGK3-mediated phosphorylation of PIKfyve and subsequent PI(3,5)P_2_ production act to regulate AMPA receptor channel expression via Rab11-dependent vesicle trafficking.

## Materials and Methods

### Molecular biology

#### cDNAs, vectors, and lipids

The following cDNAs were used: GluA1/pSGEM, SGK3/pGHJ, PIKfyve and PIKfyve(S318A)/pCDNA3.1, Rab11 and Rab11(S25N)/pCDNA3.1 (from D. Marks and E. Pagano), and myosin and myosin delRBD(del1797-1811)/pCDNA6b-His/V5 (from M.D. Ehlers). PI(3,5)P_2_-DIC8 (P4508) was purchased from Echelon Bioscience Inc. (Salt Lake City, USA).

#### cRNA synthesis

Template DNA was linearized with a suitable restriction enzyme. cRNA was synthesized from 1 µg of linearized DNA using an *in vitro* transcription kit (mMessage mMachine T7 kit, Ambion Ltd., Cambridgeshire, UK). cRNA concentrations were evaluated by photospectrometry, and transcript quality was checked by agarose gel electrophoresis.

### Purification of total RNA from brain tissue, RT-PCR and quantitative RT-PCR analysis

C57/BL6 mice were anaesthetised with ether and then decapitated. Brains were dissected in ice-cold PBS. Immediately after preparation, transverse slices (350 µm) were cut with a vibratome and allowed to recover in a custom built submerged chamber with oxygenated PBS (95%O_2_/5%CO_2_) solution at 22°–25°C for 60 min before incubation with or without 10 µM NMDA. Slices were then nitrogen-frozen and stored at −80°C.

Total RNA was purified using the RNeasy Lipid Tissue Kit from Qiagen (Qiagen, Hilden, Germany) according to the manufacturer's recommendation (RNeasy Lipid Tissue Handbook). Brain tissue was nitrogen-frozen immediately after surgery. For homogenization 1 ml of Qiazol Lysis Reagent (Qiagen) was used for each sample. For DNAse removal the on-column DNAse digestion was performed according to the manufacturer's description. Total RNA was eluted with 30 µl DEPC water. The total RNA of 4 samples were pooled, concentrated to lower volume by speed vacuum centrifugation and frozen at −80°C for storage.

For cDNA first strand synthesis, 1 µg of total RNA in 12.5 µl DEPC-H_2_O was mixed with 1 µl of oligo-dT primer (500 µg/ml, Invitrogen, Darmstadt, Germany) and heated for 2 min at 70°C. An RT mix of 2 µl 10× reaction buffer (Biolabs, Frankfurt, Germany), 1 µl dNTP mix (dATP, dCTP, dGTP, dTTP, 10 mM each, Promega, Mannheim, Germany), 0.5 µl recombinant RNase inhibitor (Roche, Mannheim, Germany), 0.1 µl M-MuLV reverse transcriptase (Biolabs, Frankfurt, Germany), and 2.9 µl DEPC-H_2_O was then added and the reaction mixture incubated for 60 min at 42°C. The reaction was stopped by heating the mixture for 5 min at 94°C. The cDNA was stored at −20°C until PCR analysis. PCR analysis was then performed with 1 µl of the reverse transcription product in a total volume of 25 µl of a PCR mix containing 22 µl of sterile H_2_O, 1 µl of primer 1 (10 pmol/µl), 1 µl of primer 2 (10 pmol/µl), and 1 puReTaq Ready-To-Go PCR bead (Amersham Biosciences, Freiburg, Germany) through 40 cycles (30 s at 94°C; 30 s at 60°C; 45 s at 72°C). The following primers were used to amplify a 315 bp stretch of the PIKfyve isoform, bridging exons 19 and 20: sense primer: 5′-CACTTGGGCACTTGCCACA-3′; antisense primer: 5′-CTCCGTCAGAAGATAGGAATG-3′. For control reaction to exclude genomic contamination the following primers were used binding in intron 19 and exon 20: sense primer: 5′-TCATCTTAAGCATATAATCA-3′; antisense primer: 5′- TCAGAGCCTCCTCTGAGCTTG-3′. PCR products were analyzed by agarose gel electrophoresis.

For quantitative RT-PCR, 2 µg of total RNA was reverse-transcribed with Superscript II Reverse Transcriptase (Invitrogen, Darmstadt, Germany). For subsequent PCRs, cDNA from 50 ng of total RNA for quantitative PCR with a LightCycler Fast Start DNA Master Plus SYBR Green I kit (Roche, Mannheim, Germany) was used for a single reaction. Real-time PCRs were performed on a Roche LightCycler (Roche, Mannheim, Germany). Analysis was done using Roche LightCycler Software (version 3.5). β-actin was used as a house-keeping/control gene. SGK3 primer sequences used were: sense primer: 5′-ATGCAGAGGGATTGTATCATGG-3′; antisense primer: 5′-TCTTGGCAGGAATCTTCAGAGC-3′. β-actin primer sequences used were: sense primer: 5′-CGTTGACATCCGTAAAGACCT-3′; antisense primer: 5′-CAAAGCCATGCCAATGTTGTCTCT-3′.

### Phosphorylation of purified PIKfyve by recombinant PKB and SGK3

Wild-type and mutant (S318A) GST-PIKfyve (amino acids 1–497) were expressed in E. coli and purified using glutathione-Sepharose beads essentially as previously described [23]. The beads (10 to 20 µL) were incubated with 20 mU of PKB or SGK3 (Upstate, Dundee, UK) in phosphorylation buffer (20 mM MOPS pH 7.2, 1 mM EDTA, 0.1% β-mercaptoethanol, 20 mM β-glycerophosphate, 10 mM MgCl_2_, 500 µM ATP (0.05MBq [γ-32P]ATP per sample)) at 30°C for 20 minutes. Sample buffer was added and the samples heated at 95°C for 5 minutes. Samples were electrophoresed on a 4–12% Bis-Tris gel and transferred to PVDF. The membrane was analysed by autoradiography and probed with anti-pS318-PIKfyve and anti-GST antibodies as previously described [23].

### Electrophysiological measurements in *Xenopus* oocytes

Oocytes of stages V-VI were surgically removed from the ovaries of *Xenopus laevis* as described elsewhere [34]. Oocytes were injected with GluA1 cRNA (4 ng/oocyte) or together with SGK3 cRNA (6 ng/oocyte) using a nanoliter injector 2000 (WPI, Berlin, Germany). Standard two-electrode voltage clamp recordings were performed 5–7 days after cRNA injection with a TurboTec 03 amplifier (npi, Tamm, Germany) and an interface DIGIDATA 1322A from Axon Instruments (CA, USA). Data analyses were done with pClamp/Clampex software 8.0 (Axon Inc., CA, USA) and Origin 6.0 software (Additive, Friedrichsdorf, Germany). Agonist solutions were prepared in ND-96 buffer (96 mM NaCl; 1.8 mM CaCl_2_; 2.0 mM KCl; 1.0 mM MgCl_2_, and 5 mM HEPES-NaOH, pH 7.2 with NaOH, all from Sigma-Aldrich, Munich, Germany). Current and voltage electrodes were filled with 3 M KCl and had resistances of 0.5–1.5 MΩ. Oocytes were held at −70 mV and agonist (300 µM glutamate; Tocris, Cologne, Germany) was applied by superfusion for 10 s at a flow rate of 10–14 ml/min.

### Isolation of cell surface proteins after biotinyl-ConA modification

To identify the fraction of receptor protein inserted in the plasma membrane, surface proteins were tagged with biotinylated ConA (Sigma-Aldrich, Munich, Germany) and isolated by streptavidin/sepharose-mediated precipitation of the biotinyl-ConA/protein complexes as described elsewere [8]. Briefly, intact oocytes were incubated in 10 µM biotinyl-ConA (Sigma, Munich, Germany) for 30 min at room temperature. At this step the biotinylated ConA binds to glycosylated plasma membrane proteins, e.g. glutamate receptors. To remove excess biotinylated ConA, oocytes were washed five times for 10 min in ND-96 buffer. After washes, 20 intact oocytes were homogenized with a Teflon pestle in H-buffer (20 µl/oocyte; 100 mM NaCl, 20 mM Tris-HCl, pH 7.4, 1% Triton X-100, plus a mixture of proteinase inhibitors (Complete; Boehringer Mannheim, Mannheim, Germany)) and were kept at 4°C for 1 hr on a rotating rod. Since exclusively intact oocytes were used for homogenization, only plasma membrane proteins, not proteins of internal membranes, were labelled. After centrifugation of the remaining homogenate for 1 min at 16,000 g, the supernatants were supplemented with 20 µl of washed streptavidin-sepharose beads (Sigma, Munich, Germany) and incubated at 4°C for 3 hrs on a rotating rod. During this step, the streptavidin beads bound to the biotinyl-ConA-plasma membrane receptor complex. The streptavidin-sepharose beads were then pelleted by a 2 min spin at 16,000 g and washed three times in H-buffer. The final pellets, containing plasma membrane receptors, were boiled in 20 µl of SDS-PAGE loading buffer (0.8 M ß-mercaptoethanol, 6% SDS, 20% glycerol, 25 mM Tris-HCl, pH 6.8, and 0.1% bromphenol blue).

### Gel electrophoresis and Western blotting

Proteins from homogenized oocytes were separated by SDS polyacrylamide gel electrophoresis and transferred to nitrocellulose filters. Blots were blocked in 1× PBS containing 5% milk powder for at least 1 hour at room temperature. For the detection of GluA1, rabbit anti-GluA1 antibody (kind gift of R. Huganir) and secondary horseradish peroxidase-conjugated donkey anti-rabbit antibody (1∶1000 dilution, Amersham Bioscience, Freiburg, Germany) were used.

### Statistical analysis

For the immunoblotting studies, representative immunoblots are shown and a quantitative assessment of plasma membrane abundance was obtained by densitometric analysis of immunoblots from similar experiments. Before pooling the results from different blots, data from each blot were expressed as a percentage of the control value (relative abundance). The combined data from all blots were then expressed as the mean ± SEM. Statistical analyses of the data were performed by Origin 6.0. Experiments were analyzed by ANOVA, and p<0.05 was considered statistically significant.

### Primary hippocampal cell culture

New-born wild type Wistar rats of post-natal day 0–2 (P0–P2) were used for cultivation of primary rat hippocampal neurons. Primary hippocampal neurons were prepared according to a similar protocol as described by Lessmann and Heumann [35]. Briefly, the hippocampi of P0–P2 new-born Wistar rats were minced in ice cold MPBS+/+ (modified phosphate-buffered saline supplemented with 0.25 mM CaCl_2_, 5.8 mM MgCl_2_, 10 mM HEPES, 1 mM sodium pyruvate, 6 µg/ml DNaseI, 1 mg/mL bovine serum albumin (BSA), 10 mM glucose, 25 U/mL penicillin, 25 µg/ml, streptomycin, 2 mM glutamine, 5 mg/mL phenol red, 4 mM NaOH) and digested with 10% trypsin in MPBS−/− (MPBS+/+ without CaCl_2_ and MgCl_2_) for 7 min while shaking at 37°C. After settling of the tissue, the supernatant (containing dissociated cells) was diluted two-fold with RPMI/10% FCS (2 mM glutamine, 10% fetal calf serum (FCS), 25 U/mL penicillin, 25 µg/ml, streptomycin, 0.00375% insulin, 5 mM glucose, 10 mM HEPES, in RPMI 1640 (PAA)) to terminate digestion. Residual tissue pieces were dissociated by trituration in MPBS−/− with cut plastic pipette tips three times. The cells were collected by centrifuging at 200×g for 10 min. The cells were dissociated in RPMI/10% FCS, seeded at a density of 200,000 cells per 35 mm gelatine-coated glass cover slip and cultivated at 37°C and 5% CO_2_. The following day, the media was exchanged against neurobasal medium (Invitrogen, Darmstadt, Germany) containing B-27 serum-free supplement (Invitrogen) (1× B27, 2 mM glutamine, 100 U/ml penicillin, 100 µg/ml streptomycin in neurobasal medium). Every second to third day the media was exchanged against fresh pre-incubated medium. The cells were used for experiments after 10–14 days *in vitro*. The media was removed and fresh full neurobasal medium without B-27 was given to the cells 2 h prior to the stimulations. Hippocampal neurons were stimulated by either 10 µM NMDA, 3 µM PIKfyve inhibitor (YM201636; Chemdea, Ridgewood, NJ, USA), 30 µM SGK3 inhibitor (EMD638683; Merck KGaA, Darmstadt, Germany), or both for 20 minutes each. Finally, the cells were fixed with 4% paraformaldehyde in PBS.

### Immunhistochemistry

For immunohistochemistry hippocampal neurons were fixed in 4% paraformaldehyde. The neurons were either incubated with 1% Triton X-100 in PBS (permeable condition) or in PBS only (non-permeable condition) for 15 min. Thereafter, cell cultures were washed three times in PBS and incubated with primary rabbit anti-GluA1 antibody (kind gift of R. Huganir) and mouse anti-PSD95 antibody (Thermo Scientific, Rockford, IL, USA, #MA1-046), or primary rabbit anti-GluA1 antibody (Alomone, Jerusalem, Israel, #AGC-004) and mouse anti- neuroligin-1 antibody (Millipore, Temecula, CA, USA, #MABN38) overnight at 4°C. PSD95 and neuroligin-1 antibodies were used as postsynaptic neuronal markers (PSD95 for intracellular and Neuroligin-1 for extracellular staining). Afterwards, to block nonspecific binding sites, cultures were treated with 10% (w/v) normal goat serum in PBS for 30 min. The samples were then reacted with the secondary antibodies for 2 h at room temperature (goat anti-mouse IgG-TRITC, T5393; goat anti-rabbit IgG-FITC, F6005, Sigma, Munich, Germany). Finally, cultures were rinsed in PBS and coverslipped in Moviol (Aventis, Hoechst, Germany). Samples were evaluated by confocal laser scanning microscopy (Zeiss LSM 510 META, Oberkochen, Germany) in the multi-tracking sequential mode set for detection of FITC and TRITC with the appropriate emission bandpass filters in combination with Zeiss 40× (Plan-Neofluoar, NA 1.3) oil immersion lenses and the Zeiss physiology kit.

### Analyses of immunhistochemistry

#### Surface expression of GluA1

The images were analyzed using ImageJ (version 1,45 s Rasband, W.S., ImageJ, U.S. National Institutes of Health, Bethesda, Maryland, USA, http://imagej.nih.gov/ij/, 1997–2011) similar as described before [36]. The staining intensity of immunostainings depends on several factors including the quality of the substrate and the background. We tried to account for these influences in our analyses. The intensities of green (GluA1 - *G_i_*) and red (neuroligin - *N_i_*) fluorescence were determined using ImageJ in a square of about 1×25 µm covering a region of a clearly identifiable dendrite and a region outside the neuron. The intensities determined outside the neurons were regarded as background and subtracted from the dendritic intensities. To account for variations in quality of the substrates, like variances in morphology, we normalized the GluA1 intensities to the neuroligin intensities by dividing *G_i_* by *N_i_*.

#### Percentage of colocalized GluA1

Using ImageJ the intensities of a selected square of about 1×25 µm were calculated for the GluA1 and neuroligin images. The results were transferred to Origin 6.0 and the profiles were overlaid. Firstly, the clearly identifiable GluA1 peaks (*Gc*) of 0.3–3 µm size indicating protein clusters were counted [36]. Secondly, the peaks/clusters where GluA1 peaks overlapped with neuroligin peaks/clusters of 0.3–3 µm sizes were counted as colocalized clusters (*cc*). The percentage of colocalized GluA1 was calculated by (*cc/Gc*)×100%.

### Electrophysiology on hippocampal slices

Seven-to- ten week old male C57/BL6 mice were anaesthetised with ether and then decapitated. Brains were dissected in ice-cold artificial cerebrospinal fluid (aCSF) (containing in mM: NaCl, 124; KCl, 4.9; KH_2_PO_4_, 1.2; MgSO_4_, 1.3; CaCl_2_, 2.5; NaHCO_3_, 25.6; and d-glucose, 10). Immediately after preparation, transverse slices (400 µm) were cut with a vibratome and allowed to recover in a submerged chamber with oxygenated aCSF solution at 22°–25°C for 1 h before recordings were commenced. Slices were then transferred to a submerged recording chamber with continuously perfused aCSF that was saturated with 95%O_2_/5%CO_2_ at 30°C. The constant flow rate was ∼1 mL/min. A bipolar stimulating electrode was positioned in the Schaffer collaterals. Recordings of field excitatory postsynaptic potentials (fEPSPs) were obtained via a monopolar platinum-tipped silver chloride electrode that was placed in the Stratum radiatum of the CA1 region.

Data were obtained by averaging five fEPSP responses generated by test-pulse stimulation at 0.025 Hz (0.2 ms stimulus duration, 16 KHz sample rate). For each time point, five evoked responses were averaged. These time-points were timed to obtain averaged responses every 5 or 15 minute intervals. The average of the first 6 time-points acquired during the first 30 min was taken as a baseline reference with which subsequent changes in evoked responses were compared and calculated as a percentage value. The slope of the field excitatory post-synaptic potential (fEPSP) was measured as the maximal slope through the five steepest points obtained on the first positive deflection of the potential. By means of input/output curve determination the maximum fEPSP slope was found for each individual animal, and all potentials employed as baseline criteria were evoked at a stimulus intensity that produced 30% of this maximum. The data were then expressed as mean percentage preinjection baseline reading ±SEM. Statistical analysis was done using analysis of variance (ANOVA) with repeated measures and significant was set to p<0.05.

The phosphoinositol-3-phosphate-5-kinase (PIKfyve) inhibitor YM201636 was dissolved in dimethylsulfoxide (DMSO) (10 mM stock). After 40 min of baseline recordings, slices were continuously perfused with YM201636 in a concentration of 2 µM. To exclude independent effects of the vehicle, in a separate experiment DMSO was applied in the same time-period as YM201636. Effects were compared to slices that were neither treated with YM201636 or DMSO.

### Ethic Statement

New-born wild type Wistar rats and wild type C57/BL6 mice of either sex were killed following protocols approved by the animal welfare officer of the Ruhr-University Bochum in accordance with the guidelines of the European Community (86/609/EEC). All efforts were made to minimize animal suffering. The method for hippocampus preparation has been described in detail elsewhere [33].

## Supporting Information

Figure S1 **Reduced synaptic GluA1 expression after treatment with a PIKfyve inhibitor.** (**A**, **B**) Representative confocal images of dendrites stained with GluA1 (green) and PSD95 (red) in controls versus NMDA-treated neurons. (**C**) Statistical analysis of colocalization of GluA1 and PSD95 revealed reduced synaptic GluA1 expression after treatment with a PIKfyve inhibitor. (**D, E, F, G**) Magnified views of representative dendritic stainings with GluA1 and PSD95 in (**D**) control versus (**E**) NMDA, (**F**) PIKfyve, (**G**) NMDA plus PIKfyve-treated neurons. The arrows indicate co-localization of GluA1 and PSD95. Scale bars, 5 µm.
(TIF)
