# Supplementary figures and images for: Identification of a Novel Signaling Pathway and Its Relevance for GluA1 Recycling
Source: PLoS One. 2012 Mar 21;7(3):e33889. doi: 10.1371/journal.pone.0033889 (PMC3309939; doi:10.1371/journal.pone.0033889)

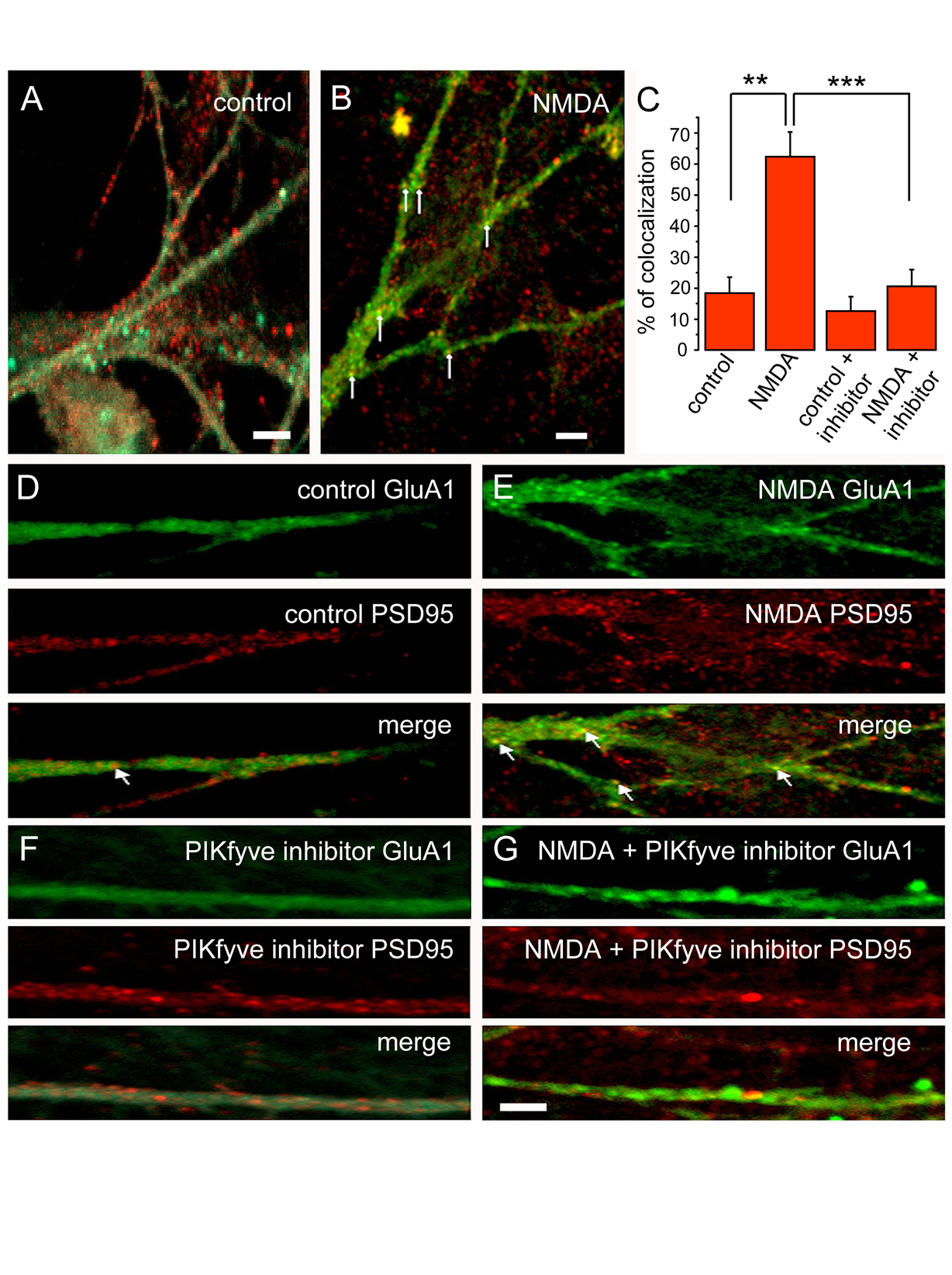

Supplement: Figure S1 — Reduced synaptic GluA1 expression after treatment with a PIKfyve inhibitor. (A, B) Representative confocal images of dendrites stained with GluA1 (green) and PSD95 (red) in controls versus NMDA-treated neurons. (C) Statistical analysis of colocalization of GluA1 and PSD95 revealed reduced synaptic GluA1 expression after treatment with a PIKfyve inhibitor. (D, E, F, G) Magnified views of representative dendritic stainings with GluA1 and PSD95 in (D) control versus (E) NMDA, (F) PIKfyve, (G) NMDA plus PIKfyve-treated neurons. The arrows indicate co-localization of GluA1 and PSD95. Scale bars, 5 µm. (TIF) [file pone.0033889.s001.tif]
